# Perceptions of Primary Care Providers on Public Health Services in Primary Care in Oman: A Qualitative Study

**DOI:** 10.7759/cureus.42208

**Published:** 2023-07-20

**Authors:** Said Al Hasani, Thamra S Al Ghafri, Ahmed S AlHarthi, Emma Gibson, Mohamed S Al Harthi

**Affiliations:** 1 Planning and Studies, Oman Ministry of Health, Muscat, OMN; 2 Primary Care, Oman Ministry of Health, Muscat, OMN; 3 College of Medicine and Health Sciences, Sultan Qaboos University, Muscat, OMN; 4 Behaviour Medicine, University of Birmingham, Dubai, ARE

**Keywords:** oman, primary care, services, public health, perceptions

## Abstract

Background

The integration of public health functions in primary care is not well-defined in the literature. This paper examines the perceptions of healthcare workers on public health services in primary care, as well as the challenges and views on strengthening the integration of public health functions in a primary care setting in Oman.

Methodology

This qualitative study (employing a face-to-face interview) was conducted in a primary healthcare setting in Muscat from January 1, 2022, to May 31, 2022. This study is based on interpretative phenomenological analysis using purposeful sampling. Participants were interviewed to answer the study questions. The targeted participants included directors and other official personnel, physicians, nurses, pharmacists, nutritionists, health educators, and lab technicians. Verbal consent was obtained from the participants before the interview, and all responses were anonymously audio recorded, transcribed verbatim, and analyzed using thematic analysis.

Results

A total of 10 primary care providers were interviewed once for 30 minutes over a two-week period. All participants were females apart from one male participant. The study included three physicians, five nurses, one pharmacist, and one nutritionist. All participants had over 10 years of experience as primary care providers at the time of the interview. The main themes were a lack of awareness of public health services in a primary care setting, challenges to practicing public health in a primary care setting, and recommendations to strengthen the integration of public health services in primary care. In general, there were inconsistent views on public health services in a primary care setting, and the interactions between the functions were not clear. Participants reported an absence of clear guidelines, training, and competencies for public health in a primary care setting. Building public health capacities and reforming the health system were highly recommended to integrate public health into primary health care.

Conclusions

Understanding how public health and primary care interact is crucial to improve population health. Building competencies and supportive health systems are required for the effective integration of public health in primary care settings.

## Introduction

The World Health Organization (WHO) defined health as a state of complete physical, mental, and social well-being and not merely the absence of disease or infirmity [1]. Since its establishment in 1948, WHO has recognized the value of addressing the well-being of an individual and the community which can be achieved by providing an equitable distribution of health to the public [2]. Public health (PH) is concerned with the state of health at the level of the population. It promotes and protects the health of people and the communities where they live, learn, work, and play [3]. The Institute of Medicine (IOM) defined PH as fulfilling society’s interest in assuring conditions in which people can be healthy [4]. PH discusses the complete health state of the community which constitutes a group of individuals. Therefore, creating a distinct boundary between the health state of an individual and the health state of a community is not always plausible, and sometimes overlaps occur that necessitate integration between the two approaches. In comparison to PH, primary health care (PHC) is a branch of medicine that addresses the majority of an individual’s health needs throughout their lifetime [5]. Traditionally, PHC is concerned with the health of an individual while PH is concerned with the health of the community. However, they share common goals in addressing issues of disease prevention and health promotion in the community [5]. The IOM reported that the integration of PHC and PH is crucial [6]. This can be achieved by broadening and deepening the roles of PHC and PH beyond the exclusive traditional concern of either the individual’s or the community’s health [5]. The integration of PH and PHC can be defined as the linkage of programs and activities to promote overall efficiency and effectiveness and achieve gains in population health [6]. It can cover a wide range of activities, including community engagement and participation, health promotion, health education, prevention activities, chronic disease management, screening, immunization, communicable disease control, information systems activities, development of best practice guidelines, needs assessment, quality assurance and evaluation, and professional education [7]. Health agencies have established different approaches to achieve the integration between PH and PHC. For example, WHO described the following five main areas of integration: (1) coordinating healthcare services for individuals; (2) applying a population perspective to clinical practice; (3) identifying and addressing community health problems; (4) strengthening health promotion and disease prevention; and (5) collaborating around policy, training, and research [8].

This study aims to explore the perception of healthcare workers on PH services in a primary care setting with regard to disease prevention and control. The specific objectives of the study are to explore (1) the perceptions of healthcare workers on PH services and competencies in primary care, (2) challenges in integrating PH in primary care, and (3) views on strengthening the integration of PH services in a primary care setting.

## Materials and methods

Study setting and conceptual framework

This study was conducted in a PHC setting in Muscat Governorate from January 1, 2022, to May 31, 2022. This qualitative research is based on the interpretative phenomenological analysis method of understanding an individual’s perception of a particular topic using purposeful sampling [9].

Study participants

All healthcare providers working in PHC institutions in Muscat Governorate, Oman were eligible to participate in the study. The targeted participants included directors and other official personnel, physicians, nurses, pharmacists, nutritionists, health educators, and lab technicians.

Methodology

Different locations and timings were utilized to conduct individual face-to-face interviews based on the convenience of the participants. All interviews were conducted in a private and quiet room and lasted approximately 30 minutes. All interviews were conducted in the English language, and a mobile audio recording application was used to record the interviews. Initially, verbal consent was obtained from the participants via a phone call before the interviews and again at the time of the interviews after explaining to them the aim and the objectives of the study and the purpose of the audio recording. Furthermore, it was explained to them that their responses would be anonymously recorded, and all audio records would be discarded at the end of the study.

Interview and topic guide

Semi-structured face-to-face interviews were conducted over an approximately one-month period. The topic guide covered the following five main areas: (1) participants’ characteristics; (2) perception of healthcare workers on the integration of PH and PHC; (3) PH core competencies in disease prevention and control in a primary care setting; (4) challenges, limitations, and strengths in the integration of PH and PHC in disease prevention and control in primary care; and (5) views on improving PH competencies in disease prevention and control in a PHC setting. Table 1 presents the topic guide and the interview questions.

**Table 1:** Semi-structured interview topic guide questions. PH = public health; PHC = primary health care

Topic	Questions
Participant’s characteristics	What is your job title? Are you currently working in PHC? How long have you been working in PHC?
Perception of healthcare workers on the integration of PH and PHC	Are you aware of any PH services provided in PHC? If yes, would you please elaborate on those services? Are you involved in providing PH services in the PHC setting? If no/yes, please elaborate. How did you identify those services as being PH rather than PHC services? In your opinion, is it important and helpful to distinguish between PH and PHC services and why?
PH core competencies in PHC	Are you aware of the core competencies of PH in a PHC setting? If yes, please elaborate. In general, what are the competencies required to strengthen the integration of PH in PHC? In your opinion, what are the PH competencies required in disease prevention and control in a PHC setting?
Challenges, limitations, and strengths in the integration of PH and PHC	With regard to disease prevention and control, what are the challenges or limitations of practicing PH and implementing competencies in a PHC setting? What are the weaknesses of building PH core competencies in a PHC setting? What are the strengths of building PH core competencies in a PHC setting?
Views on improving PH competencies in a PHC setting	How can we improve the integration of PH in PHC? How can we develop PH competencies in PHC?

Analysis

Audio records of the interviews were transcribed verbatim by a member of the research team (EG). Inductive thematic analysis was performed based on the aims and objectives of the study. The transcripts were reviewed by the research team (SH and TG) to check for accuracy and completeness. Data were coded and categorized according to the emerging themes. The final themes and subthemes were revised by an expert researcher (TG) in qualitative studies and by the research team members (SH, EG, AH, MH, and FH).

Ethical considerations

Ethical approval for the study was obtained from Muscat Regional Research Committee before conducting the study (approval number: MoH/CSR/21/25130).

## Results

A total of 10 primary care providers were interviewed once for approximately 30 minutes over a two-week period. All participants were females apart from one male participant. Participants included three physicians, five nurses, one pharmacist, and one nutritionist. All of the participants had over 10 years of experience as primary care providers at the time of the interview, and the majority held administrative positions as well such as directors, heads of units, and staff in charge with significant contributions in policy-making, service evaluation, and staff training at the primary care setting. Table 2 shows the emerging themes and subthemes.

**Table 2:** Themes and subthemes.

Themes	Subthemes
1. Awareness of public health services in a primary care setting	1.1 Inconsistent views on public health services in a primary care setting
	1.2 The interaction between public health and primary care services
	1.3 Prior sensitization to public health core competencies in a primary care setting
2. Challenges to public health practice in a primary care setting	2.1 Lack of differentiation between public health and primary care services
	2.2 Absence of clear guidelines and competencies for public health in a primary care setting
	2.3 Untrained staff in public health competencies
3. Recommendations to strengthen the integration of public health services in primary care	3.1 Development of human resources to strengthen public health capacities and skills
	3.2 Disease prevention and control as the center of public health in primary health care
	3.3 Reform the health system to integrate public health into primary health care

Awareness of public health services in a primary care setting

All participants expressed that they were aware of PH services at a PHC level, while eight out of 10 stated that they were directly involved in delivering PH programs.

Inconsistent Views on Describing Public Health Services in a Primary Care Setting

Participants expressed different views on PH and the majority were unclear about the definition of PH services. The main definition centered on community services, disease prevention, and infection control.

“Health service has two main arms, the clinical arm, which is provided to the clients at the health institutions, and the other arm where you work with the community….” P1

 “Well, when you say public health, to me, it’s combating a clinical disease like COVID-19 ... Public health is about preventing something, so combating COVID-19 is about preventing the spreading of the infection. That’s for me is public health.” P10.

The Interaction Between Public Health and Primary Care Services

Participants were confident about defining immunization and screening programs as the core of PH services in PHC. Others reported geriatric, chronic illnesses, antenatal clinics for pregnant ladies, community health services, and school health services as PH services delivered within PHC. However, few participants expressed no demarcation between PH and PHC services.

“We start just screening people for non-communicable diseases, trying to increase their awareness about the importance of preventing such diseases.” P1

“I can list them down like geriatric, chronic illnesses, immunization for children and for healthcare workers, ANC, antenatal clinics for pregnant ladies, community health services, school health services, and immunization for school students.” P6

“…for example, we have a malaria prevention program.” P8

“We know that public health, anything related to prevention, promotion of health … like a chronic disease screen program to cancer screening program.” P5

“I think it’s the same. There is no difference between public health and primary care. It should be like one service.” P2

Prior Sensitization to Public Health Core Competencies in a Primary Care Setting

Almost all participants reported no prior knowledge of PH definitions and the required competencies in PHC.

“I am not aware of public health competencies in primary care.” P1

“No, I am not aware….” P8

Challenges to public health practice in a primary care setting

Lack of Differentiation Between Public Health and Primary Care Services

Despite more than half of the participants expressing no difference between PH and PHC in real practice, more than half of the participants recognized the importance of defining both services separately while others expressed no importance.

“Without guidance, people will continue to just be interchanging both services. Maybe they do some public health services, but they don’t know that this is public health services, and vice versa.” P1

“It is very important and helpful to distinguish between them, but it should not be separated….” P6

“I think it should be as one system or so because the main objective of the primary healthcare and the public healthcare is to prevent the occurrence of any disease in the community.” P8

Absence of a Clear Vision, Guidelines, or Competencies of Public Health in a Primary Care Setting

All participants expressed a lack of proper vision, guidelines, or competencies to practice PH in PHC.

“…establishing a program without a clear vision! how would you be able to monitor it?” P1

“There must be clear policies, guidelines addressing this public health issue, and how can we integrate the public health services within the primary healthcare.” P1

Untrained Staff in Public Health Competencies

Almost half of the participants stated that PHC providers need training on providing PH services in a PHC setting. This was especially important to respond to outbreaks and emergencies.

“…we need to train the staff who is giving the service to the public or to the patients and provide the training and the materials for them….” P2

“I think the shortage of staff, one of the elements, can be. They do not have that much knowledge about the service and program that they are providing. they need more training about that, and sites of the structures of the health center, it's not also suitable to give the service.” P2

“I think we need training awareness of the healthcare provider about the types of disease that can be prevented.” P8

Recommendations to strengthen the integration of public health services in primary care

Development of Human Resources to Strengthen Public Health Capacities and Skills

Almost all participants expressed that an increase in the number of trained staff in PH is important to improve the PH services in a PHC setting. Building capacities on the management of PH programs in PHC was also highly recommended.

“We need human resources that are only dedicated to those services because they are busy with other services as well. You are overloading them with public health services and prevention programs.” P1

“We have to start by building capacity ... I think the healthcare workers are the carrier of these programs, so they have to know when to start screening patients, how to do prevention, how to raise awareness in the community….” P10

Disease Prevention and Control as the Center of Public Health in Primary Health Care

Participants had different perspectives on what PH services are to be targeted for improvement in a PHC setting. Policies that support PH programs in PHC were emphasized. Many participants highlighted the role of PH competencies in the management of outbreaks.

“Having well-structured programs to prevent mental health diseases in the community is a must. How can we have those programs without trained people and policies for those programs? What are the structures of those programs?” P1

“When it comes to prevention, I think one of the major components is patients’ activation and their engagements in whatever public health program you want to initiate….” P4

“I think primary healthcare should have some core competency in public health, especially what we faced last year and the year before with the pandemic.” P10

Reform the Health System to Integrate Public Health in Primary Health Care

Some of the participants highlighted issues related to the PHC setting, including the electronic system, providing more spaces, and offering transportation and other logistics for healthcare workers while providing PH services. These actions are essential to ensure sustainability and further PH integration in PHC. Effective and supportive health systems/models are required to support team-based interventions where the roles of physicians, nurses, and PH practitioners can be synergized.

“It should be part of our electronic system as well….” P1

“…..we don’t have that many places to provide any public health service and we need more places for that. This is regarding the structures.” P2

“….system support is one of the weaknesses because they don’t provide them, like transportation support, the other risk allowances, if anything happens to them out there in the community, as well as the ambulance services, if any emergency happens during carrying out the procedure. This is the system support.” P6.

## Discussion

The integration of PH and PHC was stressed in the 1978 Alma-Ata declaration on PHC and the 1986 Ottawa Charter for Health Promotion [10,11]. However, in practice, this integration is never optimum. This is due to the ways in which the two sectors are perceived and delivered [12].

The terms “public health” and “primary care” can be defined differently across cultures. One of the common definitions of “public health” is “the art and science of preventing disease, prolonging life, and promoting health through the organized efforts of society” [13]. This definition can very well fit the scope of PHC. Health functions and activities interchange between PH and PHC where some are entirely situated in one of the two, while others belong to both of them. Screening and immunization, for example, as well as interventions to support healthy lifestyles, are PH functions, while surveillance, planning, and evaluation are PH activities that can enhance primary care [14,15].

The results of this study are consistent with the literature regarding the integration of PHC and PH. The domains for this integration include community engagement and participation, health promotion, health education, prevention activities, chronic disease management, screening, immunization and communicable disease control, and professional education [7]. Community service, for example, was reported in this study as a core PH service. In this regard, coordination of clinical services with community services is warranted. This can be explained by combining clinical services such as prevention, diagnosis, and treatment or rehabilitation with counseling, outreach, and social programs. This model has been reported in Europe where health promotion community centers are set up within all PHC centers [12].

Another analytical tool reported in this study was the supportive policies that can address clinical epidemiology, risk assessment, and cost-effectiveness analysis to enhance practice management [8]. This approach along with proper data analysis in primary care can support the health promotion and disease prevention PH interventions.

Similar to other studies, the results of this study emphasize the importance of a supportive health system [16]. A review article on the collaboration between PHC and PH published in 2012 and covering 114 studies distinguished between (a) systemic factors that include the environment outside of the organizational policy and fit with local needs, funding and resource factors, power and control issues, and education and training; (b) organizational factors that include common agenda, knowledge and resource limitations, leadership, management and accountability issues, geographic proximity of partners, and shared protocols, tools, and information and interactional factors that support this collaboration [7]; and (c) interpersonal (or interactional) factors that include having a shared purpose [14].

During the COVID-19 pandemic, the most successful interventions were PH driven [17]. A systematic review of epidemic prediction of these interventions revealed that under different strategies, the most significant effect was in travel restrictions. There were different studies on the impact of contact tracking and social isolation, but it was considered that improving the quarantine rate and reporting rate, and the use of protective face masks were essential for epidemic prevention and control. Therefore, prevention and control as PH functions were enforced within the healthcare system to combat this emergency [17].

Additionally, PHC had a crucial role in responding to the COVID-19 pandemic as the first point of patient care. Major transform services to accommodate PH functions were experienced in relation to COVID-19. Despite being overwhelmed with guidance, they often lacked access to practical training [18]. Similar transformations were reported in Oman, where the situation was perceived by PHC workers as a new experience that challenged PHC, enforced the utilization of PH/epidemiological skills, and linked to unfavorable socio-religious and psychological events [19,20].

Finally, results from this study can be utilized to inform a PHC system reform where dimensions of PH and PHC are well structured, integrated, and synergized.

Study limitations

This study is not without limitations. First, the interviews were conducted by the principal investigator (epidemiologist) who is working in the same institution as the participants which may have introduced interviewer bias unintentionally. However, to ensure no interviewer bias, the interviewer read the questions to the interviewees exactly as they are written without interpretation of the meaning or the purpose of the questions. If the question was not understandable, it was repeated to the interviewees to allow them to respond according to their best understanding of the question. Second, no software for qualitative analysis was used which may have reduced the accuracy of the analysis. However, in our study, we conducted the analysis manually using the six phases of thematic analysis by Braun and Clarke which include familiarizing with the data, generating initial codes, searching for themes, reviewing themes, defining and naming themes, and producing the report [21].

## Conclusions

Results from this qualitative study support the global view for effective and pragmatic integration of PH in PHC. Despite reported challenges in the interaction between PH and PHC, training staff, disease prevention and control, and system reform to support PH were all highly recommended. Further interventional studies are needed to improve the interaction between PH and PHC to create synergy from their activities.
